# Analysis of oncogenic *AKT1* p.E17K mutation in carcinomas of the biliary tract and liver

**DOI:** 10.1038/sj.bjc.6604498

**Published:** 2008-07-15

**Authors:** M-O Riener, M Bawohl, P-A Clavien, W Jochum

**Affiliations:** 1 Department of Pathology, Institute of Surgical Pathology, University Hospital Zurich, Schmelzbergstrasse 12, Zurich CH-8091, Switzerland; 2 Department of Surgery, Division of Visceral and Transplantation Surgery, University Hospital Zurich, Zurich CH-8091, Switzerland

**Sir,**

We have read with great interest the article by Kim *et al* (2008), in which they report the analysis of 713 cancer tissues for somatic mutations in the *AKT1*, *AKT2* and *AKT3* genes. They detected the previously reported *AKT1* pleckstrin homology domain mutation (*AKT1* p.E17K) in 4 out of 93 (4.3%) breast carcinomas, but not in the colorectal, lung, gastric and hepatocellular carcinomas of their series (Carpten *et al*, 2007). The authors concluded that the *AKT1* p.E17K mutation should be further analysed in a wider range of cancers.

We have studied 118 carcinomas of the biliary tract and liver (11 intrahepatic and 34 extrahepatic cholangiocarcinomas, 23 gallbladder carcinomas and 50 hepatocellular carcinomas) for *AKT1* p.E17K mutations using polymerase chain reaction (PCR) and direct DNA sequencing. DNA was extracted from formalin-fixed, paraffin-embedded tumour tissues. The primers for PCR amplification and sequencing of exon 4 had the following sequence: forward 5′-CTGGCCCTAAGAAACAGCTCC-3′ and reverse 5′-CGCCACAGAGAAGTTGTTGA-3′. Reaction conditions for PCR amplification were 40 cycles of 95°C for 30 s, 60°C for 1 min and 72°C for 1 min. Polymerase chain reaction products were purified (GFX PCR DNA and Gel Band Purification Kit, Amersham Biosciences, Otelfingen, Switzerland) and analysed on an automated DNA sequencer (model 3130) using Gene Scan and SeqScape software (Applied Biosystems, Foster City, CA, USA). Nucleotide sequences were compared with the genomic sequence of *AKT1* (ENSG00000142208).

We did not observe *AKT1* p.E17K mutations in any of the 118 carcinomas of the biliary tract and liver in our series.

We have recently shown that the frequent activation of the PI3K/AKT pathway in carcinomas of the biliary tract and liver is associated with *PIK3CA* hot spot mutations in a small subset of these tumours (Riener *et al*, 2008). The low frequency of these mutations lead to the conclusion that further genetic changes have to be responsible for the activation of the PI3K/AKT pathway in these cancers. The recently identified *AKT1* p.E17K mutation appeared to be a good candidate for such a mechanism. Our results in hepatocellular carcinomas confirm the findings of Kim *et al* in a different ethnic background and suggest that cholangiocarcinoma and gallbladder carcinoma can be added to the list of cancers that lack *AKT1* p.E17K mutations.
